# CYLD Mutations, PD-L1 and HPV; A Pan-Cancer Landscape Analysis

**DOI:** 10.21203/rs.3.rs-10854026/v1

**Published:** 2026-09-11

**Authors:** Umut Dişel, Saumya Dushyant Sisoudiya, Smruthy Sivakumar, Razelle Kurzrock

**Affiliations:** Acıbadem Adana Hospital; Foundation Medicine Inc; Foundation Medicine Inc; Medical College of Wisconsin Cancer Center

## Abstract

**Background:**

*CYLD*, a tumor suppressor gene encoding a deubiquitinase enzyme, regulates tumorigenesis via NF-κB signaling. *CYLD* mutations are linked to immune evasion and PD-L1 upregulation, particularly in virus-associated cancers. We evaluated *CYLD* alterations across cancers and their correlation with PD-L1 expression. Given the established link between *CYLD* dysfunction and viral oncogenesis, we also assessed the co-occurrence of *CYLD* alterations and human papillomavirus (HPV) status.

**Methods:**

We conducted a real-world data analysis of *CYLD* alterations in 145,991 tumor samples that underwent clinical-grade comprehensive genomic profiling during routine care; PD-L1 expression via immunohistochemistry (IHC) was assessed in 10,053 specimens. The relationship between *CYLD* alterations and high-risk HPV across cancers was also evaluated.

**Results:**

*CYLD* mutations occurred in 0.57% (836/145,991) of tumors, with the highest rates in thymic carcinomas (15.4%, 34/220), anal carcinomas (11.4%, 55/479), and head and neck cancers (6.4%, 154/2,423). Among 10,053 samples with PD-L1 IHC evaluated, 62 (0.6%) harbored *CYLD* alterations. Overall, 61% (38/62) of *CYLD*-altered tumors were positive for PD-L1 IHC expression (≥ 1%), with 29% (18/62) exhibiting high PD-L1 expression (≥ 50%). In addition, 27.5% (230/836) of *CYLD*-altered tumors were HPV+, with strong enrichment in head and neck cancers (odds ratio [OR] = 7.4; p < 0.0001).

**Conclusions:**

We highlight the rarity of *CYLD* mutations across most cancers but underscore their relatively elevated prevalence in thymic carcinomas, anal carcinomas, and head and neck cancers. The observed associations between *CYLD* alterations, PD-L1 expression, and HPV positivity suggest that *CYLD* status, combined with immune and viral biomarkers, may merit exploration as a predictor of immunotherapy benefits.

## INTRODUCTION

*CYLD*, a tumor suppressor gene encoding a deubiquitinase (DUB) enzyme, plays a crucial role in tumor suppression by negatively regulating NF-κB signaling through the removal of Lys63-linked ubiquitin chains from signaling proteins. This regulation is essential in controlling inflammation and immune responses, which are key contributors to cancer progression (1-4). Germline alterations in *CYLD* are associated with CYLD cutaneous syndrome, characterized by multiple benign skin tumors, while somatic mutations have been detected across various human cancers. Recent studies have also explored the relationship between *CYLD* mutations and immune evasion mechanisms, particularly through PD-L1 upregulation, which is central to immune suppression in cancers such as human papilloma virus (HPV) positive head and neck squamous cell carcinoma (5).

The loss of CYLD function results in increased NF-κB activity and PD-L1 overexpression, which fosters immune suppression and tumor progression. This mechanism is especially relevant in cancers such as HPV-positive head and neck, cervical, and anal cancers, where *CYLD* mutations, along with NF-κB hyperactivation, contribute to immune evasion and oncogenesis (6-9). Similarly, thymic epithelial tumors have been linked to Epstein-Barr Virus (EBV)-associated oncogenesis, where immune evasion mechanisms, including PD-L1 overexpression, may play a pivotal role (10). The relevance of PD-1/PD-L1 biology in HPV-positive cancers has been further emphasized by Mishra and colleagues, who noted the growing importance of PD-1/PD-L1 checkpoint inhibitors in HPV-associated oral carcinogenesis (11).

In the current study, we analyzed *CYLD* mutations across a broad spectrum of cancers profiled using a comprehensive genomic profiling assay and explored their association with PD-L1 expression levels via immunohistochemistry (IHC). Our goal was to better understand the landscape of *CYLD* mutations and their implications for immunotherapy.

## METHODS

The study included a retrospective analyses of a cohort comprising 145,991 patients with solid tumors who received comprehensive genomic profiling (CGP) using the laboratory-developed test FoundationOne^®^ assay as part of routine clinical care. Briefly, DNA was extracted from formalin-fixed, paraffin-embedded (FFPE) tissue sections, and CGP was performed on hybridization-captured, adapter ligation-based libraries of ~ 315 cancer-related and select introns of genes commonly rearranged in cancer, as described previously (12). Analysis of sequencing data and identification of different classes of genomic alterations was carried out as described previously (12-14). The identified alteration types include short variants (base substitutions and indels), copy number alterations, and rearrangements. Variants of unknown significance were not included (14). Only one sample per patient was included in this study.

PD-L1 expression was determined using IHC performed on FFPE tissue sections using the Dako 22C3 PD-L1 antibody. PD-L1 expression was binned into three categories based on the percentage of tumor cells staining with ≥ 1 + intensity (TPS): negative (< 1%), low positive (1–49%), or high positive (≥ 50%). Data on PD-L1 expression was available for 10,053 patients.

A two-step approach was used to detect HPV in this cohort. First, a computational algorithm leveraging the sequencing coverage on a list of pathogens, designed in the hybrid capture assay, is used. Second, a *de novo* assembly of off-target sequencing reads left unmapped to the human reference genome (hg19) as described previously. These assembled contigs were competitively mapped by BLASTn (BLAST+ v2.6.0) to NCBI database of viral nucleotide sequences to detect oncoviruses, including the HPV types. A positive status was determined by contigs ≥ 80 nucleotides in length and with ≥ 97% identity to the BLAST sequence. Samples that were positive for any of the following high-risk HPV types by either approach were classified as HPV+: HPV-16, HPV-18, HPV-31, HPV-33, HPV-35, HPV-39, HPV-45, HPV-51, HPV-52, HPV-56, HPV-58, HPV-59. These annotations are for research use only. In a prior study, HPV status of samples was confirmed using p16 staining. (15-17) Approval for this study, including a waiver of informed consent and Health Insurance Portability and Accountability Act (HIPAA) waiver of authorization was obtained from the Western Institutional Review Board (WCG IRB, Protocol number: 20152817).

Prevalence of PD-L1 expression among *CYLD*-altered vs *CYLD* wild type tumors was compared using a Fisher’s exact test in the pan-solid cohort as well as in each tumor type. Co-occurrence analysis for the presence of high-risk HPV among *CYLD* altered vs unaltered tumors was also done using a Fisher’s exact test in the overall cohort as well as within each tumor type. Comparisons were done between high-risk HPV + vs HPV− cases. Cases with only low-risk HPV were excluded from this analysis; 145,719/145,991 samples were eligible for this analysis. All P-values were adjusted for multiple hypothesis correction using the Benjamini-Hochberg FDR procedure. All reported P-values in this study are after FDR correction, unless stated otherwise. Statistical significance was set at an FDR-corrected P ≤ 0.05. Statistical analyses and plotting were carried out using Python 2.7.18 (Python Software Foundation) and R 4.2.3 (R Foundation for Statistical Computing).

## RESULTS

### Prevalence of CYLD Alterations Across Cancers

*CYLD* alterations were identified in 0.57% (n = 836/145,991) of all patients with solid tumors, indicating that these alterations are relatively rare in the broader cancer population (Table 1, Fig. 1A). Among the different tumor types, *CYLD* was most commonly altered in thymic carcinomas (15.4%; N = 34/220 patients), followed by anal carcinomas (11.4%; N = 55/479 patients), and head and neck cancers (6.4%; N = 154/2,423 patients) (Table 1, Fig. 1A). Among the *CYLD* altered cases (n = 836), samples exhibited *CYLD* short variants (76.8%; n = 642/836), copy number homozygous deletions (19%; n = 156/836) and rearrangements (6%; n = 50/836), with some cases showing multiple of these alteration types **(Supplementary Table 1).** The most common short variants affecting *CYLD* were nonsense and frameshift alterations with the most common alterations being in codons 371, 722, 42, 605 and 397 (Fig. 1B, **Supplementary Table 1)**. For example, the nonsense alteration S371* was found in 30 patients in the overall cohort, followed by N722fs*13 found in 20 patients.

### Association of CYLD Alterations and High PD-L1 Expression

Among the 10,053 samples with PD-L1 expression available from IHC, 62 (0.6%) harbored *CYLD* alterations. Almost 61% (38/62) of CYLD-altered tumors were positive for PD-L1 expression (≥ 1%) compared to 44% (4,400/9,991) of CYLD-negative tumors, showing an association between *CYLD* alterations and PD-L1 expression (OR: 2.01; 95% CI: 1.2–3.5; *P* < 0.01). Notably, among tumors expressing high PD-L1 (≥ 50%), the association with *CYLD* alterations showed a similar trend but did not reach statistical significance (OR: 2.03, 95% CI: 1.0-3.9; *P* = 0.069), with 29% (18/62) of these tumors exhibiting high PD-L1 expression (Table 2, Fig. 2). In thymomas, two of the 34 *CYLD*-mutated cases also had PD-L1 expression data available, and both cases demonstrated high PD-L1 expression (≥ 50%), further supporting a possible link between CYLD dysfunction and immune checkpoint activation. In other tumor types, including anal carcinomas and head and neck cancers, we were limited by cohort size to conduct additional statistical analyses.

### Association of CYLD Alterations and high-risk HPV status

This analyses was performed for 145,719/145,991 pan-solid tumor cases which were either positive high-risk HPV or negative for any HPV. Samples that were only positive for low-risk HPV were excluded from these analysis. Of the 836 patients overall with *CYLD* alterations, 27.5% (n = 230) were also positive for high-risk HPV (Table 3). Strong co-occurrence of *CYLD* alterations and high-risk HPV + was observed pan-cancer (OR = 10.0, *P* < 0.0001) and specifically in head and neck cancers (OR = 7.4; *P* < 0.0001), non-small cell lung cancer (OR = 3.3; *P* < 0.01), and colorectal cancers (OR = 4.8; *P* < 0.01) (Table 3). In anal carcinomas, of the 479 cases, 469 were either positive for high-risk HPV or were HPV negative. Of note, almost 81% (n = 380/469) of these were high-risk HPV + and 11% (n = 50/469) had both *CYLD* alterations and were high-risk HPV+; we observed a trend of co-occurrence between HPV + and the presence of *CYLD* alterations in anal carcinomas (OR = 2.5) although it did not reach statistical significance after FDR correction (raw *P* = 0.045) (Table 3). We were limited by cohort size to conduct additional statistical analyses in other tumor types where HPV viral infections are prominent.

## DISCUSSION

Our findings demonstrate that *CYLD* alterations, while rare in the overall cancer population, are enriched in specific malignancies, particularly thymic carcinomas, anal carcinomas, and head and neck cancers. These results align with data from the TCGA and AACR Genie databases, highlighting a similar pattern of prevalence. Comparisons between our dataset and existing literature consistently emphasize the importance of *CYLD* in these cancer types (7, 8, 18-21).

Somatic *CYLD* alterations are notable across various cancers, with TCGA data showing a 1.4% prevalence across ~ 11,000 tumors (20). We similarly showed a prevalence of *CYLD* mutations in 0.57% (N = 836/145,991) of all patients with solid tumors. In our cohort, *CYLD* was most commonly altered in thymic carcinomas (15.4%; N = 34/220 patients), followed by anal carcinomas (11.4%; N = 55/479 patients), and head and neck cancers (6.4%; N = = 154/2,423 patients). In TCGA, *CYLD* alterations were found in 2.4% of thymomas and in 2.9% of head and neck cancers (19), keeping in mind that the types of patients seen in these TCGA and in our dataset may also differ, since patients sent by clinicians for molecular profiling by the FoundationOne^®^ assay often have advanced and/or refractory disease. Notably, an association exists between *CYLD* mutations and HPV-related cancers, particularly HPV-positive head and neck squamous cell cancers, cervical cancer, and anal carcinoma. Among 515 head and neck squamous cell tumors reported in TCGA, 2.9% harbored *CYLD* mutations, with a pronounced enrichment in HPV-positive cases (11%) compared to HPV-negative cases (1.7%) (16). Additional studies corroborate this trend. For example, Gillison et al. reported *CYLD* alterations in 7.4% of HPV-positive HNSCCs versus 0.6% in HPV-negative tumors, while Seiwert et al. identified *CYLD* mutations in 6% of HPV-positive and 1% of HPV-negative cases (22, 22)

Consistent with prior studies from TCGA and other datasets, our study confirmed a strong association between *CYLD* alterations and HPV positivity, with 27.5% of *CYLD*-altered tumors also harboring high-risk HPV. The enrichment was particularly striking in head and neck cancers, NSCLC, and colorectal cancers.

In another study of anal carcinomas, *CYLD* mutations were observed in 13% of cases, with 97% of these tumors associated with high-risk HPV (8). In our study, 11% of anal cancers (n = 50/469) had both *CYLD* alterations and were high-risk HPV+. These observations also underscore a potential association between *CYLD* alterations and HPV positivity in anal carcinoma, albeit limited by cohort size and warranting additional validation. It has been suggested that *CYLD* alterations, in conjunction with HPV oncoproteins, contribute to tumor progression by activating the NF-κB signaling pathway, upregulating proinflammatory cytokines, and promoting PD-L1 overexpression, thus driving immune evasion (8, 23).

*CYLD* mutations are also significantly associated with thymic epithelial tumors (TETs), particularly thymic carcinomas (24). In our study, 15.4% (34/220) of thymic carcinomas harbored *CYLD* mutations.

These alterations, along with NFKBIA mutations, are implicated in constitutive NF-κB activation and elevated PD-L1 expression, positioning *CYLD* as a molecular driver in thymic carcinoma (1).

Further studies demonstrate that low *CYLD* expression in TETs is strongly associated with increased PD-L1 expression, particularly via IFN-γ-mediated activation of the STAT1/IRF1 pathway (19). These findings suggest the potential utility of PD-1/PD-L1 inhibitors in this context (25). A study by Giaccone et al. identifies *CYLD* mutations (p.S331* and p.R850*) exclusively in responders to immunotherapy, with these mutations being associated with significantly higher PD-L1 expression (p = 0.0031) (26). Indeed, in that study, all five patients with a *CYLD* mutation had high expression of PD-L1 (p = 0.00067). Three of these five patients had a complete or partial response after pembrolizumab immunotherapy. The findings suggest that *CYLD* mutations may serve as predictors for immunotherapy response in thymic carcinoma (25, 26). Additionally, evidence suggests that EBV, a known oncogenic virus, may contribute to thymic carcinoma pathogenesis, particularly in cases with progressive malignancy. While the role of EBV in thymomas remains unclear, its association with thymic carcinomas warrants further exploration (27). Finally, our own study herein across cancers demonstrates that 61% (38/62) of *CYLD*-altered tumors were positive for PD-L1 expression (≥ 1% by IHC); indeed, almost 30% (18/62) of these tumors exhibited high PD-L1 expression (≥ 50% by IHC).

Collectively, our analysis underscores the potential importance of *CYLD* mutations in driving immune evasion mechanisms in cancers associated with viral infections, such as HPV. This study builds on prior research linking *CYLD* dysfunction to immune evasion through PD-L1 upregulation, particularly in virus-associated malignancies. The observed correlation between *CYLD* mutations and PD-L1 expression highlights the potential for combining *CYLD* mutation status with PD-L1 levels as a biomarker for patient selection for immunotherapy.

Our study has important limitations that need to be acknowledged. This study looked at genomic alterations, sequencing-based HPV status and limited cases with available PD-L1 IHC. However, unfortunately treatment information and clinical outcomes were not available in this study cohort. Future work assessing the these patterns with respect to immunotherapy exposure may help better understand the clinical implications of our findings. Importantly, PD-L1 IHC testing is more commonly used in specific tumor types which may contribute to potential biases and warrants additional interrogation. While we already stratify all the analyses by tumor type, a multivariable model in larger cohorts with additional clinicogenomic variables may help overcome some of the limitations in the univariate analysis done in this study. The number of cases with available PD-L1 IHC was limited in this cohort and the PD-L1 IHC testing patterns may show bias towards specific tumor types during routine CGP, thereby limiting our capability to perform more detailed statistical comparisons. Specifically, some subsets were very small; for instance, there were only 2 of 34 *CYLD*-mutated thymic tumors; while both had PDL IHC > 50%, larger numbers of patients require investigation. Larger studies are warranted to corroborate these findings. Other limitations include the retrospective design, possible selection bias, limited PD-L1 and HPV data, functional uncertainty for some CYLD variants and lack of treatment and outcome data”

In conclusion, our results provide a comprehensive real-world overview of *CYLD* alterations across solid tumors and their relationship with immune and viral biomarkers. The observed associations with PD-L1 expression and HPV status are consistent with emerging mechanistic evidence. This work is hypothesis-generating and represents a large-scale confirmation of prior and recent findings; future studies should delve into mechanisms underlying the observation. *CYLD* and its relationship with viral and immune markers have previously been implicated in immunoregulation; our work did not include clinical correlations, but further support the concept that the role of *CYLD* warrants further exploration vis a vis its clinical impact. Specific next steps include analysis of ICI-treated *CYLD* cohorts, along with looking at PD-L1 and HPV status and future prospective validation of CYLD as a predictive marker .

## Figures and Tables

**Figure 1:**
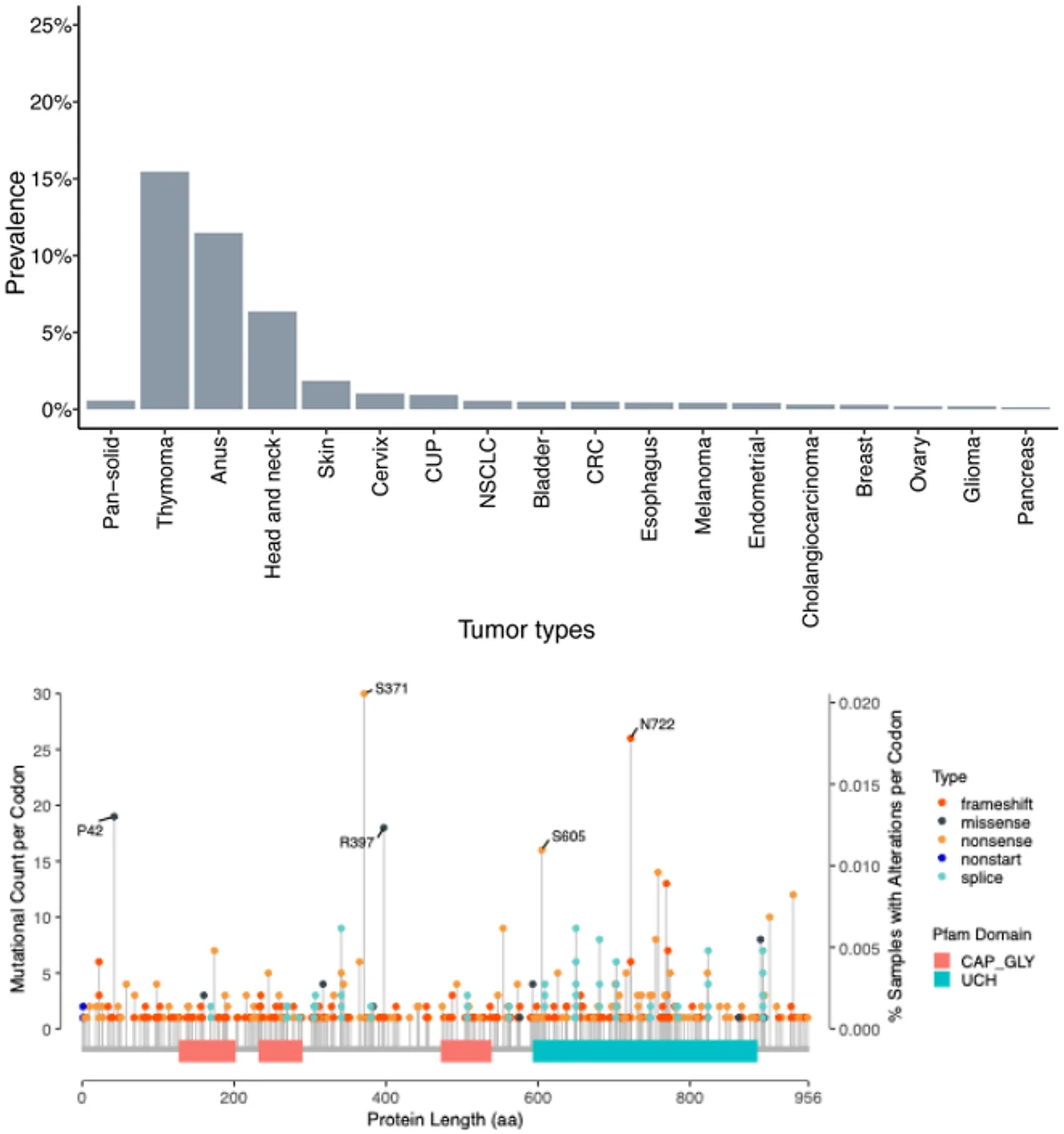
Spectrum of *CYLD* alterations across tumor types. A. Proportion of samples with *CYLD* alterations in the pan-solid cohort and across specific tumor types. Tumor types with at least 10 cases with alterations in *CYLD* are shown. B. Lollipop plot showing the distribution of *CYLD* short variants detected pan-tumor. **Abbreviations:** CUP: Unknown primary carcinoma; NSCLC: Non-small cell lung carcinoma; CRC: Colorectal cancer.

**Figure 2:**
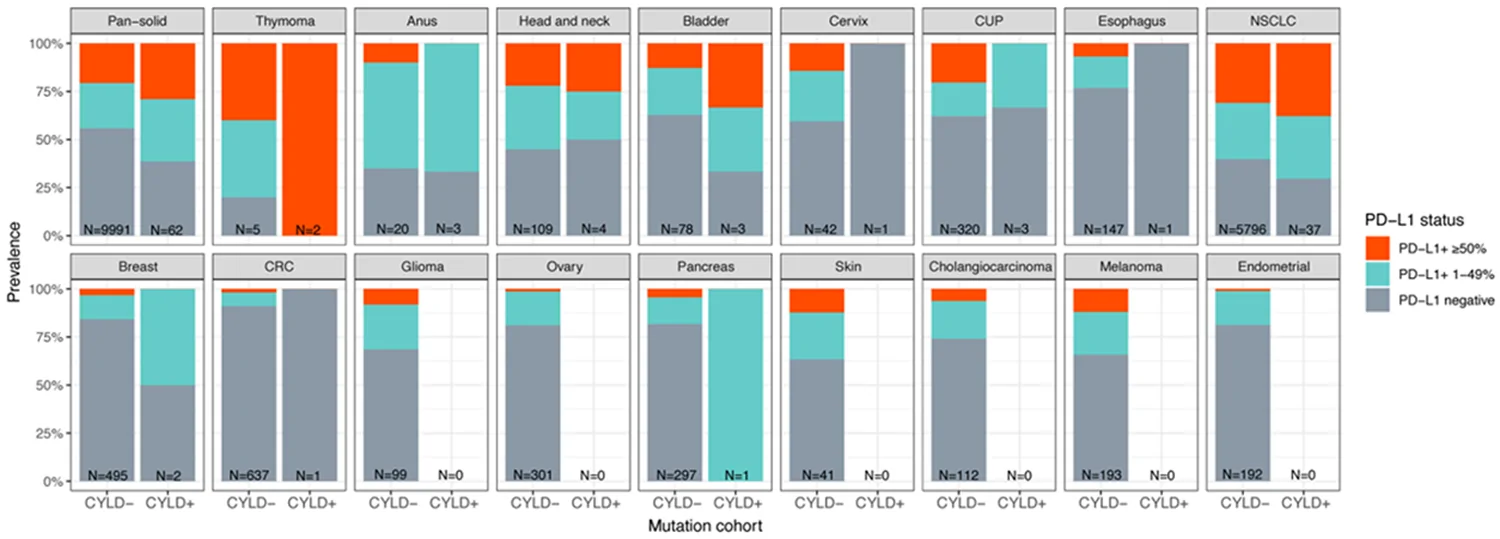
Breakdown of PD-L1 immunohistochemistry staining by *CYLD* alteration status across tumor types The proportion of samples stratified by PD-L1 status (negative, PD-L1+ 1-49%, PD-L1+ ≥50%) among cases with (CYLD+) or without (CYLD-) *CYLD*alterations in the pan-solid tumor cohort and across specific tumor types. Tumor types with at least 10 cases with assessed PD-L1 status are shown. PD-L1 status was determined using IHC using the Dako 22C3 PD-L1 antibody. **Abbreviations:** CUP: Unknown primary carcinoma; NSCLC: Non-small cell lung carcinoma; CRC: Colorectal cancer.

**Table 1:** Prevalence of pathogenic *CYLD* alterations across tumor types (N = 145,991 cancers)

Tumor type	Number of total samples	Number of samples with altered *CYLD*	*CYLD* alteration prevalence
Pan-solid cancers	145991	836	0.57%
Thymic carcinoma	220	34	15.45%
Anus	479	55	11.48%
Head and neck	2423	154	6.36%
Skin	753	14	1.86%
Cervix	1067	11	1.03%
Unknown primary carcinoma (CUP)	8543	80	0.94%
Non-small cell lung carcinoma (NSCLC)	29986	166	0.55%
Bladder	2224	11	0.49%
Colorectal (CRC)	17467	86	0.49%
Esophagus	3587	16	0.45%
Melanoma	4411	19	0.43%
Endometrial (uterine)	4066	17	0.42%
Cholangiocarcinoma	3167	10	0.32%
Breast	15252	44	0.29%
Ovary	8270	16	0.19%
Glioma	6752	13	0.19%
Pancreas	8188	11	0.13%

**Table 2:** Breakdown of PD-L1 immunohistochemistry expression prevalence by *CYLD* mutation status across top tumor types.*

Tumor type	Mutation cohort	PD-L1 available (n)	PD-L1 negative (n)	PD-L1+ (1–49%) (n)	PD-L1+ (≥ 50%) (n)
Pan-solid	*CYLD+*	62	24	20	18
Pan-solid	*CYLD−*	9991	5591	2342	2058
Thymic carcinoma	*CYLD+*	2	0	0	2
Thymic carcinoma	*CYLD−*	5	1	2	2
anus	*CYLD+*	3	1	2	0
anus	*CYLD−*	20	7	11	2
head and neck	*CYLD+*	4	2	1	1
head and neck	*CYLD−*	109	49	36	24
bladder	*CYLD+*	3	1	1	1
bladder	*CYLD−*	78	49	19	10
cervix	*CYLD+*	1	1	0	0
cervix	*CYLD−*	42	25	11	6
Unknown primary carcinoma (CUP)	*CYLD+*	6	4	2	0
Unknown primary carcinoma (CUP)	*CYLD−*	320	199	56	65
esophagus	*CYLD+*	1	1	0	0
esophagus	*CYLD−*	147	113	24	10
Non-small cell lung carcinoma (NSCLC)	*CYLD+*	37	11	12	14
Non-small cell lung carcinoma (NSCLC)	*CYLD−*	5796	2305	1702	1789
breast	*CYLD+*	2	1	1	0
breast	*CYLD−*	495	417	62	16
colorectal (crc)	*CYLD+*	1	1	0	0
colorectal (crc)	*CYLD−*	637	580	45	12
glioma	*CYLD+*	0	0	0	0
glioma	*CYLD−*	99	68	23	8
ovary	*CYLD+*	0	0	0	0
ovary	*CYLD−*	301	244	53	4
pancreas	*CYLD+*	1	0	1	0
pancreas	*CYLD−*	297	243	41	13
skin	*CYLD+*	0	0	0	0
skin	*CYLD−*	41	26	10	5
cholangiocarcinoma	*CYLD+*	0	0	0	0
cholangiocarcinoma	*CYLD−*	112	83	22	7
melanoma	*CYLD+*	0	0	0	0
melanoma	*CYLD−*	193	127	43	23
endometrial	*CYLD+*	0	0	0	0
endometrial	*CYLD−*	192	156	34	2

* *CYLD* showed statistically significant co-occurrence with PD-L1 expression in the pan-solid cohort (OR: 2.01; *P* < 0.014; 95% CI: 1.2–3.5). The other tumor types did not reach statistical significance or had limited samples for statistical analyses.

**Table 3:** Breakdown of HPV+ prevalence by *CYLD* mutation status across top tumor types

Tumor type	Mutation cohort	HPV+ (n)	HPV− (n)	OR	Lower 95% CI	Upper 95% CI	P (raw)	P (FDR)
Pan-solid	*CYLD+*	230	606	10.0	8.6	11.7	< 0.0001	< 0.0001
Pan-solid	*CYLD−*	5282	139601					
Thymic carcinoma	*CYLD+*	0	34	NA	NA	NA	NA	NA
Thymic carcinoma	*CYLD−*	4	182					
anus	*CYLD+*	50	5	2.5	0.9	8.4	0.045	0.11
anus	*CYLD−*	330	84					
head and neck	*CYLD+*	107	47	7.4	5.1	10.8	< 0.0001	< 0.0001
head and neck	*CYLD−*	532	1723					
bladder	*CYLD+*	0	11	0	0	12.2	1	1
bladder	*CYLD−*	71	2134					
cervix	*CYLD+*	8	3	1.5	0.4	8.9	0.75	1
cervix	*CYLD−*	669	378					
Unknown primary carcinoma (CUP)	*CYLD+*	28	52	10.1	6.1	16.5	< 0.0001	< 0.0001
Unknown primary carcinoma (CUP)	*CYLD−*	427	8014					
esophagus	*CYLD+*	0	16	0	0	12.3	1	1
esophagus	*CYLD−*	75	3492					
Non-small cell lung carcinoma (NSCLC)	*CYLD+*	13	153	3.3	1.7	5.8	0.0003	0.001
Non-small cell lung carcinoma (NSCLC)	*CYLD−*	748	29022					
breast	*CYLD+*	1	43	1.0	0.03	6.2	0.62	1
breast	*CYLD−*	330	14853					
colorectal (crc)	*CYLD+*	8	78	4.8	1.9	9.9	0.0005	0.0017
colorectal (crc)	*CYLD−*	366	16992					
glioma	*CYLD+*	0	13	0	0	17.4	1	1
glioma	*CYLD−*	126	6603					
ovary	*CYLD+*	0	16	0	0	12.3	1	1
ovary	*CYLD−*	172	8064					
pancreas	*CYLD+*	0	11	0	0	16.6	1	1
pancreas	*CYLD−*	193	7975					
skin	*CYLD+*	3	11	5.5	0.9	21.9	0.03	0.08
skin	*CYLD−*	35	702					
cholangiocarcinoma	*CYLD+*	1	9	5.3	0.1	39.1	0.19	0.40
cholangiocarcinoma	*CYLD−*	65	3090					
melanoma	*CYLD+*	0	19	0	0	9.3	1	1
melanoma	*CYLD−*	100	4285					
endometrial	*CYLD+*	0	17	0	0	8.9	1	1
endometrial	*CYLD−*	109	3934					

A. Proportion of samples with *CYLD* alterations in the pan-solid cohort and across specific tumor types. Tumor types with at least 10 cases with alterations in *CYLD* are shown. B. Lollipop plot showing the distribution of *CYLD* short variants detected pan-tumor.

## Data Availability

All data analyzed during this study are included in this manuscript.

## References

[1] Sun S.C. CYLD: a tumor suppressor deubiquitinase regulating NF-kappaB activation and diverse biological processes. Cell Death Differ 17, 25–34 (2010). 10.1038/cdd.2009.43 PMC5848464 19373246

[2] Patel SP, Kurzrock R. PD-L1 Expression as a Predictive Biomarker in Cancer Immunotherapy. Mol Cancer Ther. 2015;14(4):847–56. 10.1158/1535-7163.MCT-14-0983 25695955

[3] Bevins NJ, Okamura R, Montesion M, Adashek JJ, Goodman AM, Kurzrock R. Tumor Infiltrating Lymphocyte Expression of PD-1 Predicts Response to Anti-PD-1/PD-L1 Immunotherapy. J Immunother Precis Oncol. 2022;5(4):90–97. 10.36401/JIPO-22-9 PMC9714418 36483582

[4] Fountzilas E, Kurzrock R, Vo HH, Tsimberidou AM. Wedding of Molecular Alterations and Immune Checkpoint Blockade: Genomics as a Matchmaker. J Natl Cancer Inst. 2021;113(12):1634–1647. 10.1093/jnci/djab067 PMC9890928 33823006

[5] Schrank T. P., Prince A. C., Sathe T., Wang X., Liu X., Alzhanov D. T., Burtness B., Baldwin A. S., Yarbrough W. G., & Issaeva N. (2022). NF-κB over-activation portends improved outcomes in HPV-associated head and neck cancer. Oncotarget, 13(707–722). 10.18632/oncotarget.28232 PMC9131933 35634245

[6] Hajek M., Sewell A., Kaech S., Burtness B., Yarbrough W. G., & Issaeva N. (2017). TRAF3/CYLD mutations identify a distinct subset of human papillomavirus-associated head and neck squamous cell carcinoma. Cancer, 123(10), 1778–1790. 10.1002/cncr.30570 PMC5419871 28295222

[7] Williams EA, Montesion M, Alexander BM, Ramkissoon SH, Elvin JA, Ross JS, Williams KJ, Glomski K, Bledsoe JR, Tse JY, Mochel MC. CYLD mutation characterizes a subset of HPV-positive head and neck squamous cell carcinomas with distinctive genomics and frequent cylindroma-like histologic features. Mod Pathol. 2021;34(2):358–370. 10.1038/s41379-020-00672-y PMC7817524 32892208

[8] Williams E.A., Montesion M., Sharaf R. CYLD-mutant cylindroma-like basaloid carcinoma of the anus: a genetically and morphologically distinct class of HPV-related anal carcinoma. Mod Pathol 33, 2614–2625 (2020). 10.1038/s41379-020-0584-2 PMC7685972 32461623

[9] Prasad M, Cheng AT, Mayer M, Genomic enrichment and functional impact of TP53 and CYLD alterations in recurrent and metastatic HPV-associated head and neck cancer. Clin Cancer Res. Published online January 28, 2026. 10.1158/1078-0432.CCR-25-3972 PMC12959380 41603724

[10] Jovanovic D., Markovic J., Ceriman V., Peric J., Pavlovic S., & Soldatovic I. Correlation of genomic alterations and PD-L1 expression in thymoma. J Thorac Dis 12, 7561–7570 (2020). 10.21037/jtd-2019-thym-13 PMC7797854 33447447

[11] Mishra A. PD-1/PD-L1 biology and immunotherapy in HPV-positive oral cancers. Future Oncol 13, 1907–1909 (2017). 10.2217/fon-2017-0115 28829196

[12] Frampton GM, Fichtenholtz A, Otto GA, Wang K, Downing SR, He J, Schnall-Levin M, White J, Sanford EM, An P, Sun J, Juhn F, Brennan K, Iwanik K, Maillet A, Buell J, White E, Zhao M, Balasubramanian S, Terzic S, Richards T, Banning V, Garcia L, Mahoney K, Zwirko Z, Donahue A, Beltran H, Mosquera JM, Rubin MA, Dogan S, Hedvat CV, Berger MF, Pusztai L, Lechner M, Boshoff C, Jarosz M, Vietz C, Parker A, Miller VA, Ross JS, Curran J, Cronin MT, Stephens PJ, Lipson D, Yelensky R. Development and validation of a clinical cancer genomic profiling test based on massively parallel DNA sequencing. Nat Biotechnol. 2013;31(11):1023–31. 10.1038/nbt.2696 PMC5710001 24142049

[13] Milbury CA, Creeden J, Yip WK, Smith DL, Pattani V, Maxwell K, Sawchyn B, Gjoerup O, Meng W, Skoletsky J, Concepcion AD, Tang Y, Bai X, Dewal N, Ma P, Bailey ST, Thornton J, Pavlick DC, Frampton GM, Lieber D, White J, Burns C, Vietz C. Clinical and analytical validation of FoundationOne^®^CDx, a comprehensive genomic profiling assay for solid tumors. PLoS One. 2022;17(3):e0264138. 10.1371/journal.pone.0264138 PMC8926248 35294956

[14] Knepper TC, Montesion M, Russell JS, The Genomic Landscape of Merkel Cell Carcinoma and Clinicogenomic Biomarkers of Response to Immune Checkpoint Inhibitor Therapy. Clin Cancer Res. 2019;25(19):5961–5971. 10.1158/1078-0432.CCR-18-4159 PMC6774882 31399473

[15] Williams Erik A “Vulvar Squamous Cell Carcinoma: Comprehensive Genomic Profiling of HPV+ Versus HPV− Forms Reveals Distinct Sets of Potentially Actionable Molecular Targets.” JCO precision oncology vol. 4 PO.19.00406. 16 Jun. 2020, 10.1200/PO.19.00406 PMC7446361 32923875

[16] Knepper TC, Montesion M, Russell JS, The Genomic Landscape of Merkel Cell Carcinoma and Clinicogenomic Biomarkers of Response to Immune Checkpoint Inhibitor Therapy. Clin Cancer Res. 2019;25(19):5961–5971. 10.1158/1078-0432.CCR-18-4159 PMC6774882 31399473

[17] Lechner M, Frampton GM, Fenton T, Targeted next-generation sequencing of head and neck squamous cell carcinoma identifies novel genetic alterations in HPV + and HPV− tumors. Genome Med. 2013;5(5):49. Published 2013 May 29. 10.1186/gm453 PMC4064312 23718828

[18] Umemura S., Zhu J., Chahine J. J., Kallakury B., Chen V., Kim I. K., Zhang Y. W., Goto K., He Y., & Giaccone G. (2020). Downregulation of CYLD promotes IFN-γ mediated PD-L1 expression in thymic epithelial tumors. Lung Cancer, 147, 221–228. 10.1016/j.lungcan.2020.07.018 32738418

[19] Zhu X, Meng X, Mochel MC, Wiliams EA, Dresser K, Tomaszewicz K, Hutchinson L, Bledsoe JR. CYLD-mutated anal squamous cell carcinoma: an uncommon entity associated with cylindroma-like morphology and adverse clinical features. Hum Pathol. 2025 Apr 10.1016/j.humpath.2025.105765 40180056

[20] Cui Z., Kang H., Grandis J. R., & Johnson D. E. (2021). CYLD Alterations in the Tumorigenesis and Progression of Human Papillomavirus–Associated Head and Neck Cancers. Molecular Cancer Research, 19(1), 14–24. 10.1158/1541-7786.MCR-20-0565 PMC7840145 32883697

[21] Gillison ML, Akagi K, Xiao W, Jiang B, Pickard RKL, Li J, Swanson BJ, Agrawal AD, Zucker M, Stache-Crain B, Emde AK, Geiger HM, Robine N, Coombes KR, Symer DE. Human papillomavirus and the landscape of secondary genetic alterations in oral cancers. Genome Res. 2019;29(1):1–17. 10.1101/gr.241141.118 PMC6314162 30563911

[22] Seiwert TY, Zuo Z, Keck MK, Khattri A, Pedamallu CS, Stricker T, Brown C, Pugh TJ, Stojanov P, Cho J, Lawrence MS, Getz G, Brägelmann J, DeBoer R, Weichselbaum RR, Langerman A, Portugal L, Blair E, Stenson K, Lingen MW, Cohen EE, Vokes EE, White KP, Hammerman PS. Integrative and comparative genomic analysis of HPV-positive and HPV-negative head and neck squamous cell carcinomas. Clin Cancer Res. 2015;21(3):632–41. 10.1158/1078-0432.CCR-13-3310 PMC4305034 25056374

[23] An J, Mo D, Liu H, Veena MS, Srivatsan ES, Massoumi R, Rettig MB. Inactivation of the CYLD deubiquitinase by HPV E6 mediates hypoxia-induced NF-kappaB activation. Cancer Cell. 2008;14(5):394–407. 10.1016/j.ccr.2008.10.007 PMC2651888 18977328

[24] Zhang X, Zhang P, Cong A, Feng Y, Chi H, Xia Z, Tang H. Unraveling molecular networks in thymic epithelial tumors: deciphering the unique signatures. Front Immunol. 2023;14:1264325. 10.3389/fimmu.2023.1264325 PMC10577431 37849766

[25] He Y., Ramesh A., Gusev Y., Bhuvaneshwar K., & Giaccone G. (2021). Molecular predictors of response to pembrolizumab in thymic carcinoma. Cell Reports Medicine, 2(15_suppl), 100392. 10.1016/j.xcrm.2021.100392 PMC8484507 34622229

[26] Giaccone G., Kim C., Thompson J., McGuire C., Kallakury B., Chahine J. J., (2018). Pembrolizumab in patients with thymic carcinoma: a single-arm, single-centre, phase 2 study. The Lancet Oncology, 19(3), 347–355. 10.1016/S1470-2045(18)30062-7 PMC10683856 29395863

[27] Zhao L., Ding J.Y., Tao Y.L., Zhu K., & Chen G. (2023). Detection of Epstein–Barr virus infection in thymic epithelial tumors by nested PCR and Epstein–Barr-encoded RNA ISH. Infectious Agents and Cancer, 18(1), 37. 10.1186/s13027-023-00497-9 PMC10257281 37296417

